# Effect of Huanglongbing or Greening Disease on Orange Juice Quality, a Review

**DOI:** 10.3389/fpls.2018.01976

**Published:** 2019-01-22

**Authors:** Bruno M. Dala-Paula, Anne Plotto, Jinhe Bai, John A. Manthey, Elizabeth A. Baldwin, Rhuanito S. Ferrarezi, Maria Beatriz A. Gloria

**Affiliations:** ^1^Food Biochemistry Laboratory, Department of Food, College of Pharmacy, Federal University of Minas Gerais, Belo Horizonte, Brazil; ^2^United States Department of Agriculture, Agricultural Research Service, Horticultural Laboratory, Fort Pierce, FL, United States; ^3^Indian River Research and Education Center, Institute of Food and Agricultural Sciences, University of Florida, Fort Pierce, FL, United States

**Keywords:** *Candidatus* Liberibacter asiaticus, Valencia, Hamlin, flavor, bitter compounds

## Abstract

Huanglongbing (HLB) or citrus greening is the most severe citrus disease, currently devastating the citrus industry worldwide. The presumed causal bacterial agent *Candidatus* Liberibacter spp. affects tree health as well as fruit development, ripening and quality of citrus fruits and juice. Fruit from infected orange trees can be either symptomatic or asymptomatic. Symptomatic oranges are small, asymmetrical and greener than healthy fruit. Furthermore, symptomatic oranges show higher titratable acidity and lower soluble solids, solids/acids ratio, total sugars, and malic acid levels. Among flavor volatiles, ethyl butanoate, valencene, decanal and other ethyl esters are lower, but many monoterpenes are higher in symptomatic fruit compared to healthy and asymptomatic fruit. The disease also causes an increase in secondary metabolites in the orange peel and pulp, including hydroxycinnamic acids, limonin, nomilin, narirutin, and hesperidin. Resulting from these chemical changes, juice made from symptomatic fruit is described as distinctly bitter, sour, salty/umami, metallic, musty, and lacking in sweetness and fruity/orange flavor. Those effects are reported in both Valencia and Hamlin oranges, two cultivars that are commercially processed for juice in Florida. The changes in the juice are reflective of a decrease in quality of the fresh fruit, although not all fresh fruit varieties have been tested. Earlier research showed that HLB-induced off-flavor was not detectable in juice made with up to 25% symptomatic fruit in healthy juice, by chemical or sensory analysis. However, a blend with a higher proportion of symptomatic juice would present a detectable and recognizable off flavor. In some production regions, such as Florida in the United States, it is increasingly difficult to find fruit not showing HLB symptoms. This review analyzes and discusses the effects of HLB on orange juice quality in order to help the citrus industry manage the quality of orange juice, and guide future research needs.

## Introduction

Huanglongbing (HLB) is a citrus disease that has profoundly changed the size and shape of worldwide citrus production, and the negative effects keep impacting the industry as the disease continues to spread throughout the various citrus growing regions of the world (Gottwald et al., 2012). Practically all commercial citrus species and cultivars are vulnerable to HLB. The disease has an array of symptoms which can be detected anywhere on the plant, from the roots to the leaves, changing the chemical characteristics, and sensory attributes of the fruit (Bové, 2006; Baldwin et al., 2010, 2018; Dala Paula et al., 2018). In this review, the effects of HLB on orange juice quality are described based on the current scientific literature.

## Worldwide Consumption and Production of Fresh Oranges and Orange Juice

Orange juice is the most widely consumed fruit juice in the world (Markestrat., 2016). Brazil is the world's largest orange producer and is forecasted to reach production levels of ~17.3 million tons for the 2017/2018 season. China is foreseen to be the second largest producer with 7.3 million tons followed by the European Union−6.3 million tons, the United States (US)−3.6 million tons, and Egypt−3.2 million tons (USDA-FAS Foreign Agricultural Service, 2018a). For the 2018–2019 season, Brazilian commercial orange production is predicted to decrease 27% due to high temperatures in October 2017 and stress from the previous production cycle (USDA-FAS Foreign Agricultural Service, 2018b). American commercial orange production is projected to drop 23% due to several reasons including the damage by Hurricane Irma in September 2017 added to the presence of HLB in Florida, and unfavorably hot weather in California (USDA-FAS Foreign Agricultural Service, 2018a).

Currently, citrus producers in many countries are facing serious problems with the emergence of the HLB disease (Teixeira et al., 2008; Bassanezi et al., 2009, 2011; Spreen and Zansler, 2015). HLB was responsible for the decrease in the production of oranges for processing in the United States from 7.98 to 2.22 billion tons (72.2% reduction) from 2007–08 to 2017–18. The fresh fruit market also decreased from 2.10 to 1.70 billion tons (20.5% reduction) during the same time interval. This market was less impacted than the rest of the citrus industry because, in the United States, around 90% of the oranges produced in Florida, the state with the largest prevalence of HLB, are processed while California supplies oranges for the fresh market (USDA-NASS, 2018). Singerman et al. (2017) reported an increase from $2.89 to $9.34 (3.2 times) of the price of a box of orange since HLB had been detected in the United States.

## A Brief Historical Background of Huanglongbing Outbreaks

Huanglongbing means “yellow dragon disease” in Chinese, and is also known as citrus greening (Halbert and Manjunath, 2004). HLB is considered one of the most severe citrus diseases in the world and, consequently, a serious problem for the citrus processing industry. The disease affects nearly all varieties of citrus, with grapefruit, sweet oranges, some tangelos, and mandarins being the most susceptible and limes, lemons, sour oranges, and trifoliate oranges the least (Abdullah et al., 2009).

**Figure 1:**
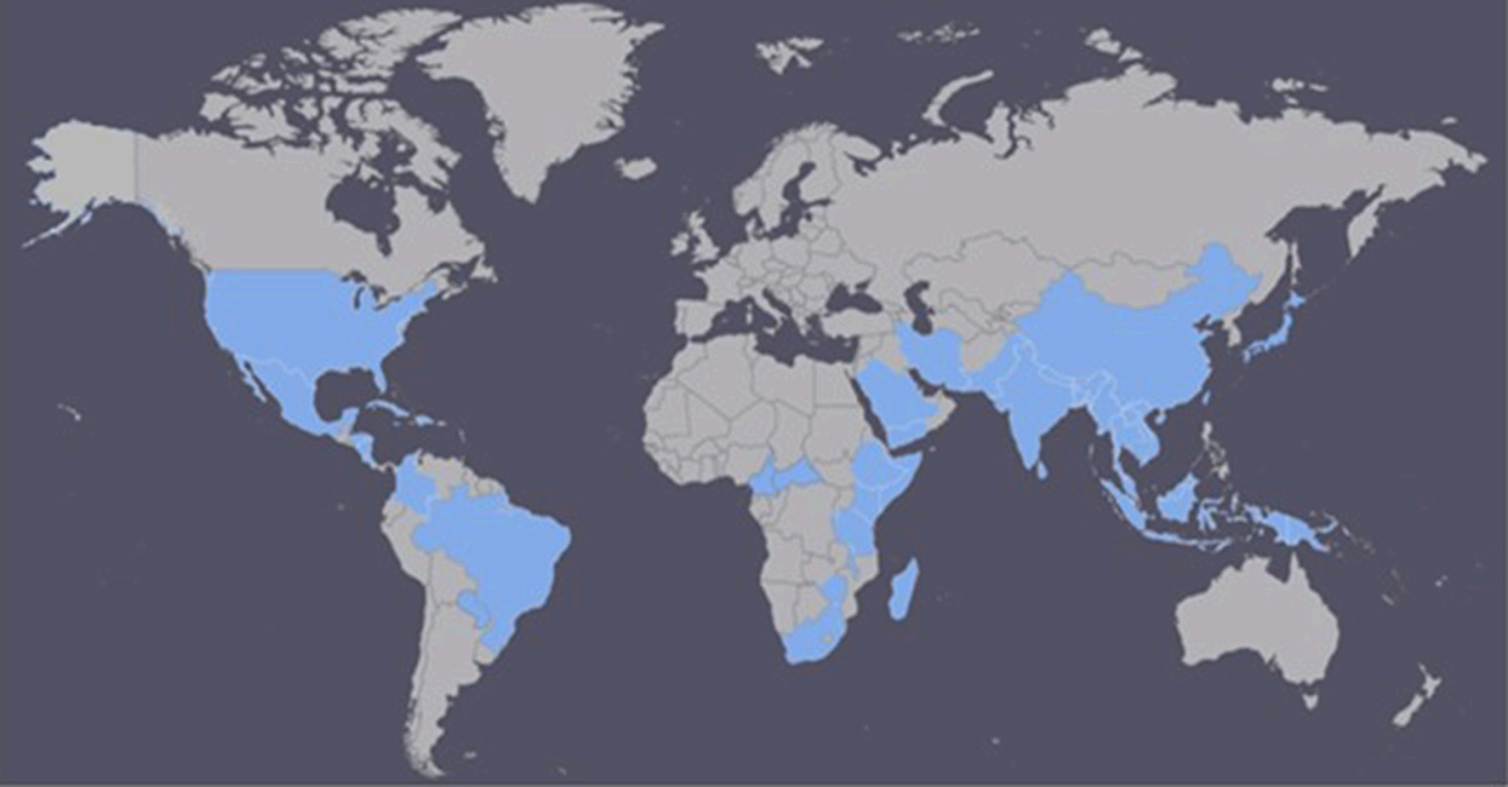
Countries currently affected by Huanglongbing (HLB) disease (adapted from CABI, 2017; EPPO, 2017).

It is difficult to determine where HLB originated. However, there is information suggesting that HLB was responsible for India's citrus dieback during the eighteenth century (Capoor, 1963; da Graça, 2008). Initially, researchers believed that the tristeza virus was the leading cause of the citrus dieback in India, but after a thorough survey, HLB was determined to be the primary cause (Fraser and Singh, 1968; da Graça, 2008). In China, HLB has been reported since 1919 and described by Reinking (1919) as the citrus yellow shoot disease (Bové, 2006). In 1937, the African variation was reported for the first time in South Africa (Van der Merwe and Andersen, 1937), and it was later linked to chromium and manganese toxicity. It was also associated with the leaf mottling citrus disease in the Philippines in the 1960's (Fraser et al., 1966; McClean and Schwarz, 1970). Currently, the disease has spread to more than 50 countries in Africa, Asia, Oceania, and the Americas (South, North and Central Americas, and the Caribbean; Figure 1; CABI, 2017; EPPO, 2017).

The first case of HLB in the Americas was reported in the state of São Paulo, Brazil in 2004 (Coletta-Filho et al., 2004; Teixeira et al., 2005a). However, in a survey conducted in São Paulo, just 6 months after HLB had been reported in Brazil, 46 cities stated having infected trees, suggesting that HLB had been present for almost 10 years (Bové, 2006). A year later, in August 2005, symptoms of the disease were recognized in Florida, United States; in 2007 in Cuba; in 2008 in the Dominican Republic; and in 2010 in Mexico (Coletta-Filho et al., 2004; Halbert, 2005; Llauger et al., 2008; Matos et al., 2009; NAPPO North American Plant Protection Organization, 2010). Currently, HLB is present in all Florida citrus-growing counties (Baldwin et al., 2010), in California, Georgia, Louisiana, South Carolina, and Texas (CABI, 2017; EPPO, 2017). As the severity of HLB increases, premature fruit drop becomes a growing problem which has contributed to declining yields in Florida, especially during the last few years (Chen et al., 2016). In Brazil, the States of São Paulo, Minas Gerais, and Paraná have reported the presence of HLB, with São Paulo being the most affected state. In India and China, HLB has spread to around 25 and 11 provinces, respectively (Table S in Supplementary Material; CABI, 2017; EPPO, 2017).

## Causal Agents and Vectors of Huanglongbing

It is well established that Huanglongbing is associated with the presence of the gram-negative bacteria genus *Candidatus* Liberibacter (*C*L). Three species are known to cause the symptoms of HLB: *C*L asiaticus (*C*Las), *C*L americanus (*C*Lam), and *C*L africanus (*C*Laf). The Asian and the American species can be transmitted by the psyllid *Diaphorina citri* Kuwayama (Hemiptera: Psyllidae), commonly called Asian citrus psyllid (ACP), and the African species by the insect *Trioza erytreae* (Hemiptera: Triozidae; Bové, 2006). Although HLB was first reported in Brazil and the US 15 years ago, the psyllid vector was reported in São Paulo and Florida as early as 1942 and 1998, respectively (Bové, 2006; Tansey et al., 2017).

*C*Lam was the most prevalent bacteria species in Brazil in 2005, which initially affected more than 90% of the infected trees, decreasing to 60% in 2007. During this period, there was an increase in *C*Las infection, from 5 to 35% of the infected trees, while a combined infection remained practically the same at 5% (Coletta-Filho et al., 2007; Gasparoto et al., 2012). Among HLB bacteria, *C*Laf is sensitive to heat and to dry weather and thrives between 20 and 25°C, while the other species are heat tolerant and thrive at higher temperatures (Catling, 1969; Cheraghian, 2013). These observations might explain why *C*Laf is not present in hot and humid tropical and subtropical climates.

As *C*Las has been difficult to culture *in vitro*, its recommended detection methods was by quantitative real-time polymerase chain reaction (qPCR) targeting the 16S rDNA gene (Teixeira et al., 2005b; Li et al., 2006).

## Symptoms of Huanglongbing and its Impact on Orange Trees

In the early stages of the disease, it is difficult to make a clear diagnosis. McCollum and Baldwin (2017) noted that HLB symptoms are more apparent during cooler seasons, more so than in warmer months. It is uncertain how long a tree can be infected before showing the symptoms of the disease but, when it eventually becomes symptomatic, symptoms appear on different parts of the tree. Infected trees generally develop some canopy thinning, with twig dieback and discolored leaves, which appear in contrast to the other healthy or symptomless parts of the tree. The symptomatic leaves can be normal-sized, showing yellow coloration or a blotchy-mottle or they can be small, upright and show a variety of chlorotic patterns resembling those induced by zinc or other nutritional deficiencies (McClean and Schwarz, 1970; da Graça, 1991; Albrecht et al., 2016; McCollum and Baldwin, 2017). The root systems are poorly developed, showing very few fibrous roots, likely due to nutrient starvation (da Graça, 1991; Batool et al., 2007).

Symptomatic trees display excessive starch accumulation in the aerial plant parts, one of the predominant biochemical responses to HLB, due to the upregulation of glucose-phosphate transport, which is involved with the increased entrance of glucose into this pathway (Martinelli and Dandekar, 2017). It has been suggested that accumulation of starch in the leaves is also the result of decreased degradation and impaired transport which results in an inefficient partitioning of photoassimilates among mature citrus leaves, roots, and young leaves. This unbalance in sugar transport and accumulation would affect sugar content in fruit. The starch indefinitely remains in the aerial plant parts; it does not degrade, even during the night cycles, resulting in root starvation, severe health decline, and death of trees (Etxeberria et al., 2009; Fan et al., 2010; Zheng et al., 2018).

Along with the color changes and starch accumulation in symptomatic leaves, there are also changes in the secondary metabolite profiles. HLB affects the amounts of hydroxycinnamic acids and flavonoids in infected leaves, resulting in lower levels of vicenin-2, apigenin-*C*-glucosyl-*O*-xyloside, 2”-xylosylvitexin, luteolin rutinoside, and isorhoifolin compared to healthy leaves. While healthy leaves contain only trace levels of limonin glucoside, infected leaves contain levels of 300 ± 22 μg/mL (Manthey, 2008). Proline and other amino-acids were found in greater amounts in leaves showing symptoms of infection, and sugar metabolism was also affected (Cevallos-Cevallos et al., 2012; Albrecht et al., 2016).

According to studies of infected orange fruit, HLB-symptomatic oranges are reduced in size, sometimes asymmetric, and contain small, brownish/black aborted seeds which can be seen when the orange is sectioned perpendicularly to the fruit axis. The orange peel turns green with an inversion of colors: the fruit turns from green to yellow/orange in the peduncular end while the stylar end remains green. In a healthy orange, the color change first starts at the stylar end, progressing only later to the peduncular area. HLB causes fruits to drop prematurely, resulting in a 30–100% yield reduction, and, ultimately, premature death of the tree. Tree mortality can occur several months to years after infection (McClean and Schwarz, 1970; da Graça, 1991; Bové, 2006; Batool et al., 2007; Bassanezi et al., 2011; Liao and Burns, 2012).

**Figure 2:**
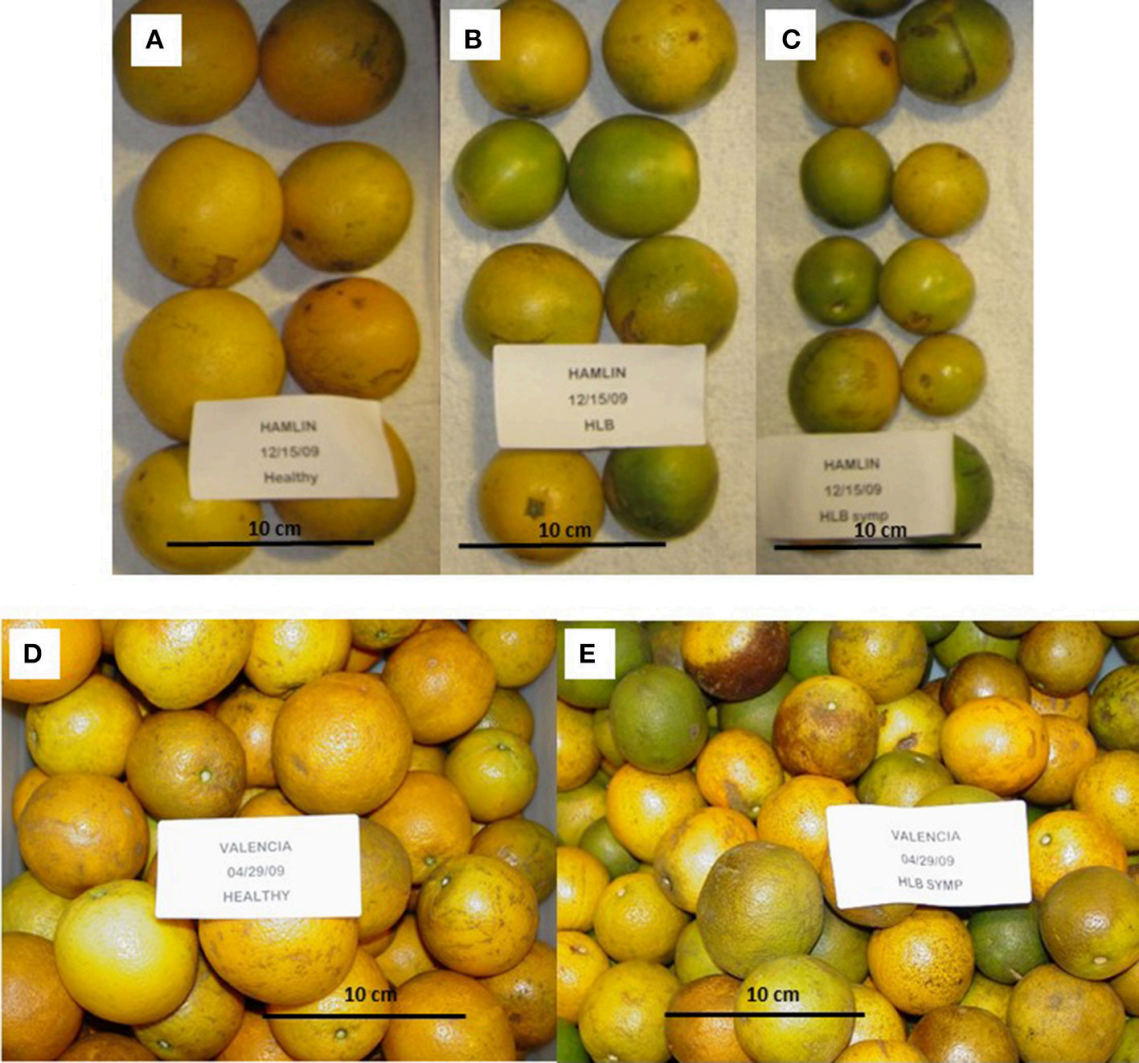
Size and color differences between fruit affected by Huanglongbing (HLB). **(A)** Hamlin healthy; **(B)** Hamlin HLB-asymptomatic; **(C)** Hamlin HLB-symptomatic; **(D)** Valencia healthy; **(E)** Valencia HLB-symptomatic (Photography by the authors).

HLB symptomatic fruit from infected trees are smaller in diameter compared to asymptomatic fruit from infected and healthy trees, which have similar diameter (Table 1, Figure 2). Even though most of these symptomatic fruit do not make it to processing due to premature drop or elimination by sizing equipment (McCollum and Baldwin, 2017; Baldwin et al., 2018), more are entering the processing stream as there is not enough normal sized fruit. The weight and juice content of symptomatic oranges are diminished compared to asymptomatic and healthy oranges, which are similar (Table 1). Most of the studies were performed with Valencia and Hamlin oranges (Liao and Burns, 2012; Massenti et al., 2016; Baldwin et al., 2018), and also with two strains of Valencia, and Hamlin, Westin and Pera varieties (Bassanezi et al., 2009).

**Table 1:** Changes in diameter, weight, and juice content of fruit affected by Huanglongbing.

**References**	**Orange sample**		**Fruit parameters**		
	**Harvest time**	**Status or conditions**	**Diameter (cm)**	**Weight (g)**	**Juice (g/100 g)**
**VALENCIA ORANGE JUICE**					
Bassanezi et al., 2009^I^	Blend of different harvests^1^	HLB-AS	7.3*a*	208.1a	50.0*a*
		HLB-SY	5.9*b*	118.9b	44.6*b*
Liao and Burns, 2012^II^	April 2009	Healthy	7.4*a*	208.5a	53.2*a*
		HLB-AS	7.7*a*	214.5 a	52.9*a*
		HLB-SY	5.8*b*	122.3 b	46.1*b*
Massenti et al., 2016^III^	March and May 2013^2^	Healthy	–	183b	58.9*a*
		HLB-AS		208a	57.8*ab*
		HLB-SY		115c	55.5*b*
Baldwin et al., 2018^IV^	April 2015	Healthy	6.7*ab*	–	–
		Healthy-R	6.9*a*	
		Healthy-D	7.0*a*	
		HLB-SY-R	6.2*c*	
		HLB-SY-D	6.4*bc*	
**HAMLIN ORANGE JUICE**					
Bassanezi et al., 2009^I^	Blend of different harvests^3^	HLB-AS	6.9*a*	173.1 a	42.2*a*
		HLB-SY	6.1*b*	128.6b	39.3*b*
Liao and Burns, 2012^II^	December 2007	Healthy	7.2*a*	194.3a	52.1*a*
		HLB-AS	6.9*a*	196.6a	49.9*a*
		HLB-SY	5.3*b*	109.9b	48.8*a*
Baldwin et al., 2018^IV^	December 2014	Healthy	6.8*abc*	–	–
		Healthy-R	7.1*ab*	
		Healthy-D	7.3*a*	
		HLB-SY-R	6.7*bc*	
		HLB-SY-D	6.4*c*	
	January 2015	Healthy	6.9*a*	
		Healthy-R	6.9*a*	
		Healthy-D	6.9*a*	
		HLB-SY-R	6.1*b*	
		HLB-SY-D	6.2*b*	

*AS, asymptomatic; SY, symptomatic; Healthy, fruit harvest from healthy not shaken trees; Healthy-R, fruit harvest from healthy shaken trees (healthy-retain); Healthy-D, healthy fruit that dropped to the ground upon shaking the trees (healthy-drop); HLB-R, fruit retained on shaken HLB affected-trees; HLB-D, fruit that dropped from HLB affected-trees*.

1 Blend of oranges harvested on September 2004, July and October 2005 and August 2007;

2 Blend of oranges harvested on March and May 2013;

3 *Blend of oranges harvested on July 2007, June and July 2008*.

*Values from the same reference with the same letter within columns and same harvest time are not significantly by the following statistical analysis (^I^Student t-test with the probability of error estimated to be lower than 0.000; ^II^Duncan's multiple range test p ≤ 0.01; ^III^Tukey's test at p ≤ 0.05; ^IV^ANOVA and Tukey's test at p ≤ 0.05)*.

HLB potentially causes trees to be more susceptible to other pests including citrus longhorned beetle (*Anoplophora chinensis* Forster) attacks. In advanced cases of HLB infection, a combination of citrus longhorned beetles and *Phytophthora* fungi is common (Halbert and Manjunath, 2004; Batool et al., 2007).

## Huanglongbing Control and Mitigation of its Symptoms

Current management strategies focus on vector control, avoiding the spread of infection, or management of infected trees. The success of individual or combined approaches depends on the infestation level. In regions where disease incidence is low, the most common practices are avoiding the spread of infection by removal of symptomatic trees, protecting grove edges through intensive monitoring, use of pesticides, and biological control of the vector ACP. The management of infected trees includes enhanced nutrition by foliar sprays of readily absorbable nutrients and phytohormones, or regulating soil pH to enhance nutrient uptake, and precision irrigation based on soil moisture sensing and needs of HLB-affected trees (Stansly et al., 2010; Albrecht et al., 2012; Martini et al., 2016; Zheng et al., 2018). However, the control of HLB is still difficult, especially if bacteria are widespread and their vectors are well established. Diseased trees in abandoned citrus groves act as abundant sources of *C*Las inoculation and insect vectors, and this has been a particularly prevalent problem in Florida. The most effective control strategy has been to remove infected trees in an area and then replant with *C*Las-free trees (Abdullah et al., 2009). Current recommendations are that control of the psyllid vector should be done as soon as its presence is noticed in citrus groves, even in regions free of HLB (McCollum and Baldwin, 2017).

Another area-wide pest management approach to control the ACP and reduce the likelihood of resistance is the Citrus Health Management Areas (CHMAs) (Jones et al., 2013). According to Singerman and Useche (2016), CHMAs coordinate insecticide application to control the ACP spreading across area-wide neighboring commercial citrus groves as part of a plan to address the HLB disease. The intensifying insecticide application also creates environmental and public health concerns and side-effects to specific fauna, as the arthropod (Monzo and Stansly, 2017). Singerman and Page (2016) indicated that CHMAs enhance grower's profitability when all growers involved participated in the program.

Covered, protected production fields have been tested as an alternative for fresh citrus production in Florida. These protected systems work by physically excluding ACP from the enclosed grove therefore preventing contact between the ACP and trees. One of the main advantages is the reduced reliance upon frequent insecticide sprays to control psyllids (Ferrarezi et al., 2017a). Anti-psyllid screen houses and container-grown cultivation allow rapid young plant growth, thus playing important roles in developing new citrus production systems aimed at vector-free environments (Ferrarezi et al., 2017b).

Florida growers have been using foliar nutritional spray products that often contain macro- and micro-nutrients to compensate for lack of nutrient assimilation due to the disease, and compounds that are believed to activate “systemic acquired resistance” pathways in plants (such as salicylic acid) to increase tree defense response (Masuoka et al., 2011; Baldwin et al., 2012a). The benefits of this approach to disease management in the field have been criticized because the inoculum remains after application. Unfortunately, this perceived method of managing HLB potentially contributed to the proliferation of the disease in Florida after farmers stopped eliminating their infected trees. Unless the vector is thoroughly controlled, the spread of HLB to other orchard trees and neighboring farms is inevitable (Timmer et al., 2011; Gottwald et al., 2012). In an evaluation of the effect of nutritional spray treatments on fruit quality, Hamlin oranges from treated trees had the same off-flavor as oranges from trees that did not receive the treatment, whereas Valencia oranges were notably sweeter. Nutritional treatments did not consistently result in less pathogen DNA for either variety (Baldwin et al., 2012a). The implementation of combined nutrient programs and insecticide treatments has been studied and the results suggest that the beneficial effect of increased orange juice quality may have been cumulative, only manifesting later on (Baldwin et al., 2017; Plotto et al., 2017).

In addition to foliar nutritional sprays, plant growth regulators were tested, unsuccessfully, to reduce HLB-associated fruit drop (Albrigo and Stover, 2015). Incidentally, it was found that orange fruit showing HLB symptoms were also contaminated with *Lasiodiplodia theobromae* (diplodia), generally a postharvest pathogen, but which induced greater abscission zone in symptomatic fruit (Zhao et al., 2015). A direct correlation between diplodia and ethylene production at the fruit abscission zone was established, and the use of pre-harvest fungicides reduced fruit drop (Zhao et al., 2016). However, HLB-infected fruit with a greater abscission zone (i.e., fruit that are more readily prone to drop on the ground) had generally lower quality than fruit harvested from the same trees but with lesser abscission zone (Baldwin et al., 2018). The difference in quality was due to lower total sugars and high bitter limonoids, and was more pronounced in early-harvested Hamlin. The strategy of reducing fruit drop by reducing diplodia infection might have its benefit in delaying harvest to reduce the negative effect of HLB on fruit quality.

## Fresh Fruit and Orange Juice Quality Affected by *Candidatus* Liberibacter Asiaticus

To better understand the influence of HLB on the chemical and physicochemical characteristics of orange juice, it is important to consider the factors which may affect them, such as variety, harvest date, location, maturity, and the presence of pulp in the juice. In general, variations due to harvest date are more pronounced compared to variation due to the disease (Bassanezi et al., 2009; Baldwin et al., 2010; Plotto et al., 2010). As the season progresses, the peel color of a healthy orange becomes less green and more orange, juice content declines, sugars and soluble solids content (SSC) increase and titratable acidity (TA) and citric acid decrease (Bai et al., 2016).

### Sensory and Chemical Composition Changes of HLB-Affected Fruit

#### Peel Color

As peel color often determines the attractiveness of an orange to the consumer, the effects of HLB on this important characteristic are of great concern within the fresh fruit citrus industry. Symptomatic oranges from HLB-affected trees (HLB-SY) are greener or less orange in peel color compared to asymptomatic oranges from HLB-affected (HLB-AS) or from HLB-unaffected trees (healthy). Several studies investigated changes in peel color due to infection by *C*Las. A less orange-colored peel was reported in symptomatic Hamlin fruit (Baldwin et al., 2010, 2018; Liao and Burns, 2012). However, variation in peel color of Valencia oranges depended on harvest date and year (Baldwin et al., 2010, 2018; Liao and Burns, 2012; Massenti et al., 2016) suggesting that Valencia orange may be less prone to peel color changes due to HLB. Valencia fruit has naturally more color than Hamlin and, therefore, HLB effect on peel color would be less visible.

#### Sugar and Organic Acids

The physicochemical characteristics of oranges play a vital role in determining the quality of the orange juice produced. There is no general agreement among available results in the scientific literature regarding pH due to *C*Las infection. The pH of orange juice from HLB-infected trees were either higher, lower, or similar compared to juice made with oranges from uninfected trees (Plotto et al., 2008, 2010; Raithore et al., 2015; Dala Paula et al., 2018).

TA, SSC, and SSC/TA tend to be similar in juice from asymptomatic HLB-AS and healthy oranges. However, a few studies reported differences, although small, in SSC/TA between HLB-AS and healthy Valencia and Hamlin orange juices (Baldwin et al., 2010; Dagulo et al., 2010; Massenti et al., 2016; Hung and Wang, 2018). Juice from HLB-SY fruit usually presents the highest TA, and the lowest SSC and SSC/TA in Valencia, Hamlin (Tables 2 and 3), Westin and Pera orange juices (Bassanezi et al., 2009). Recent studies reported variation among fruit affected by the disease, with higher SSC in juice from HLB-SY Hamlin (Baldwin et al., 2018; Hung and Wang, 2018) and Valencia (Baldwin et al., 2018) and a higher SSC/TA in juice from HLB-SY Hamlin compared to juice from healthy fruit (Hung and Wang, 2018). Recently, uninfected trees are difficult to find in Florida, which explains why in the Hung and Wang (2018) study, Hamlin healthy oranges were from young 2-year old trees grown under protective screens while HLB-SY or HLB-AS oranges were obtained from older field-grown trees, making the comparison not as accurate as if trees were of the same age and growing conditions. SSC/TA, a parameter commonly used as a fruit quality index, tends to increase at later harvest dates and is more heavily affected by harvest time and orange cultivar than HLB infection status (Baldwin et al., 2010). Among the orange cultivars investigated, evaluation of the effects of HLB predominantly addresses Valencia oranges.

**Table 2:** Physicochemical characteristics of Valencia orange juice made with healthy fruit and fruit at different stages of HLB infection.

**References**	**Orange juice sample**		**Physicochemical characteristics**			
	**Harvest time**	**Status or conditions**	**pH**	**TA (g/100 mL)**	**SSC (^**°**^Brix)**	**SSC/TA**
**VALENCIA ORANGE JUICE**						
Plotto et al., 2008^I^	July 2006	Healthy FJ	4.62 b	0.64 a	12.0 a	18.8 b
		HLB FJ	4.78 a	0.54 b	11.3 a	21.0 b
		Healthy JWP	4.60 b	0.63 a	11.6 a	18.3 b
		HLB JWP	4.74 b	0.47 c	10.1 b	21.6 a
Bassanezi et al., 2009^II^	Blend of different harvests^1^	HLB-AS	–	1.22 b	9.6 a	8.3 a
		HLB-SY		1.75 a	8.0 b	4.8 b
Baldwin et al., 2010^I^	March 2007	Healthy	–	0.82 a	10.7 a	13.2 a
		HLB-AS		0.84 a	10.3 a	12.5 a
	April 2007	Healthy		0.68 a	10.1 a	15.1 a
		HLB-AS		0.72 a	9.7 a	13.6 a
	May 2007	Healthy		0.57 a	10.6 a	18.6 a
		HLB-AS		0.54 a	9.6 b	18.0 a
	June 2007	Healthy		0.43 a	11.0 a	25.8 a
		HLB-AS		0.41 a	10.1 b	24.8 a
Dagulo et al., 2010^III^	April 04, 2008	Healthy	–	–	–	13.7 a
		HLB-AS				10.8 b
		HLB-SY				5.10 c
	April 18, 2008	Healthy				14.8 a
		HLB-AS				13.0 b
		HLB-SY				5.57 c
	May 23, 2008	Healthy				18.2 b
		HLB-AS				21.5 a
		HLB-SY				9.8 c
Plotto et al., 2010^IV^	April 2008	Healthy	3.78	0.89	14.5	16.2
		HLB-AS	3.68	1.05	14.7	14.1
	June 2008	Healthy	4.37	0.42	12.0	28.7
		HLB-AS	4.27	0.46	13.2	28.4
Liao and Burns, 2012^V^	April 2009	Healthy	–	0.85 b	11.6 a	13.5 a
		HLB-AS		0.85 b	11.2 a	13.1 a
		HLB-SY		0.91 a	9.3 b	10.2 b
Slisz et al., 2012^IV^	May 2007	Healthy	–	0.54	10.6	19.5
		HLB-AS		0.52	9.6	18.5
	June 2007	Healthy		0.40	10.8	27.3
		HLB-AS		0.38	9.7	25.7
		HLB-SY		0.69	6.9	10.1
Raithore et al., 2015^III^	April 2009	Healthy	4.17 a	0.62 b	12.2 a	19.7 a
		HLB-SY	3.81 a	1.14 a	11.6 a	10.2 b
Massenti et al., 2016^III^	March + May 2013	Heatlhy	–	0.72 b	12.4 a	11.0 a
		HLB-AS		0.75 b	12.2 a	10.4 b
		HLB-SY		1.22 a	8.5 b	4.5 c
Baldwin et al., 2018^IV^	April 2015	Healthy	4.20	0.68	11.6	17.1
		HLB-SY	4.12	0.75	11.8	15.9
Dala Paula et al., 2018^VI^	March 2013	Healthy	4.35 a	0.72 b	10.5 a	14.6 a
		HLB-SY	3.86 b	0.94 a	9.6 b	10.1 b

TA, titratable acidity; SSC, soluble solids content; FJ, filtered juice; JWP, juice with pulp; AS, asymptomatic; SY, symptomatic; Healthy-R, fruit harvest from healthy not shaken trees; Healthy-R, fruit harvest from healthy shaken trees (healthy-retain); Healthy-D, healthy fruit that dropped to the ground upon shaking the trees (healthy-drop); HLB-R, fruit retained on shaken HLB affected-trees; HLB-D, fruit that dropped from HLB affected-trees.

1 Blend of oranges harvested on September 2004, July and October 2005, and August 2007;

*Values from the same reference with the same letter within columns and same harvest time do not differ in disease status, according to statistical analysis (^I^Fisher's test significant difference test at p ≤ 0.05; ^II^Student t-test with the probability of error estimated to be lower than 0.000; ^III^ANOVA and Tukey's test p ≤ 0.05; ^IV^not applicable; ^V^Duncan's multiple range test p ≤ 0.01; ^VI^ANOVA and Tukey's test p ≤ 0.05 for SSC, and p ≤ 0.001 for TA and SSC/TA)*.

**Table 3:** Physicochemical characteristics of Hamlin orange juice made with healthy fruit and fruit at different stages of HLB infection.

**References**	**Orange juice sample**		**Physicochemical characteristics**			
	**Harvest time**	**Status or conditions**	**pH**	**TA (g/100 mL)**	**SSC (^**°**^Brix)**	**SSC/TA**
**HAMLIN ORANGE JUICE**						
Bassanezi et al., 2009^I^	Fruits of different harvests^1^	HLB-AS	–	0.76 a	9.6 a	13.1 a
		HLB-SY		0.91 b	8.9 b	10.7 b
Baldwin et al., 2010^II^	December 2007	Healthy	–	0.49 a	7.8 a	16.0 a
		HLB-AS		0.50 a	7.6 a	15.3 a
	February 2008	Healthy		0.59 a	11.6 a	19.8 b
		HLB-AS		0.50 a	10.4 b	22.0 a
Plotto et al., 2010^III^	February 2008	Healthy	4.19	0.50	11.9	23.8
		HLB-AS	4.17	0.52	11.4	22.1
Liao and Burns, 2012^IV^	December 2007	Healthy	–	0.75 a	11.3 a	15.1 a
		HLB-AS		0.80 a	11.5 a	14.3 ab
		HLB-SY		0.78 a	9.1 b	11.7 b
Raithore et al., 2015^V^	January 2009	Healthy	4.22 a	0.52 a	11.4 a	21.7 a
		HLB-SY	4.22 a	0.52 a	11.3 a	21.7 a
Baldwin et al., 2018^III^	December 2014	Healthy	4.37	0.41	9.2	22.5
		HLB-SY	3.82	0.44	8.8	21.9
	January 2015	Healthy	4.37	0.42	11.2	26.7
		HLB-SY	4.28	0.46	11.6	25.7
Hung and Wang, 2018^V^	December 2016 + January 2017	Healthy	–	0.87 b	7.4 c	8.5 c
		HLB-AS		0.92 a	9.9 a	10.8 a
		HLB-SY		0.96 a	8.9 b	9.3 b

*TA, titratable acidity; SSC, solid soluble content; AS, asymptomatic; SY, symptomatic; Healthy-R, fruit harvest from healthy not shaken trees; Healthy-R, fruit harvest from healthy shaken trees (healthy-retain); Healthy-D, healthy fruit that dropped to the ground upon shaking the trees (healthy-drop); HLB-R, fruit retained on shaken HLB affected-trees; HLB-D, fruit that dropped from HLB affected-trees*.

1 *Blend of oranges harvested on July 2007, June and July 2008*.

*Values from the same reference with the same letter within columns and same harvest time do not differ in disease status, according to statistical analysis (^I^Student t-test with the probability of error estimated to be lower than 0.000; ^II^Fisher's test significant difference test at p ≤ 0.05; ^III^not applicable; ^IV^Duncan's multiple range test p ≤ 0.01; ^V^ANOVA and Tukey's test p ≤ 0.05)*.

Glucose, fructose, and sucrose were quantified in orange juice from HLB-infected trees and compared with juice from oranges from uninfected trees. In the early studies, glucose and fructose either did not vary, or slightly decreased upon the effect of disease status in fruit (Plotto et al., 2008; Baldwin et al., 2010; Slisz et al., 2012; Raithore et al., 2015; Table 4). Only recent studies reported a significant increase of glucose and fructose content in juice from HLB-SY fruit compared with healthy oranges (Baldwin et al., 2018; Dala Paula et al., 2018). On the other hand, sucrose and total sugar contents decreased in juice made with oranges from HLB-affected trees in most studies, and more notably, in juices from HLB-SY Valencia and Hamlin oranges. The change in sugars in HLB-SY fruit reflects the disruption in the plant carbohydrate metabolism reported in leaves of citrus affected by HLB (Fan et al., 2010), as well as the impaired sugar transport due to the disease (Liao and Burns, 2012; Chin et al., 2014; Zheng et al., 2018). An increase in cell-wall invertase was observed in HLB-infected leaves resulting in a decrease in sucrose content (Fan et al., 2010). Cell-wall invertase is a glycoprotein enzyme generally found in developing sink organs (roots and fruits) responsible for the hydrolysis of sucrose into glucose and fructose. Asymptomatic (HLB-AS and healthy) oranges can have sucrose contents ~2.5 times higher than that of symptomatic fruit (Slisz et al., 2012). In addition, Fan et al. (2010) suggested that *C*Las prefers to use fructose causing an accumulation of glucose and sucrose, which are metabolic resources but also signaling components that interfere through feedback inhibition on photosynthesis and contribute to HLB's yellowing leaf mottle symptoms. Poiroux-Gonord et al. (2013) also demonstrated an increase in sucrose content in the pulp of oranges next to leaves submitted to photooxidative stress despite the fact that the studied “Navelate” orange trees were not infected by *C*Las and, consequently, had no blocking or impaired transportation of the phloem sap as one of the different mechanisms attributable to the *C*Las (Hijaz et al., 2016).

**Table 4:** Contents of sugars and acids of Valencia and Hamlin orange juice made with healthy fruit and fruit at different stages of HLB infection.

**References**	**Orange juice sample**		**Sugars (g/100 mL)**				**Organic acids (g/100 mL)**	
	**Harvest time**	**Status or conditions**	**Glucose**	**Fructose**	**Sucrose**	**Total sugars**	**Citric acid**	**Malic acid**
**VALENCIA ORANGE JUICE**								
Plotto et al., 2008^I^	July 2006	Healthy FJ	2.8 a	1.9 a	4.3 a	–	0.52 a	0.13 a
		HLB FJ	2.8 a	1.9 a	4.1 a		0.45 b	0.10 b
		Healthy JWP	2.6 ab	1.8 ab	4.1 ab		0.48 ab	0.11 b
		HLB JWP	2.5 b	1.7 b	3.7 b		0.40 c	0.09 c
Baldwin et al., 2010^I^	March 2007	Healthy	1.9 a	1.9 a	4.9 a	8.7 a	–	–
		HLB-AS	1.9 a	1.9 a	4.7 a	8.6 a	
	April 2007	Healthy	1.9 a	2.0 a	5.2 a	9.1 a	
		HLB-AS	1.7 a	1.8 a	4.4 b	8.0 b	
	May 2007	Healthy	2.0 a	2.0 a	5.5 a	9.5 a	
		HLB-AS	1.8 b	1.9 a	4.8 b	8.5 b	
	June 2007	Healthy	2.0 a	2.0 a	5.6 a	9.7 a	
		HLB-AS	1.8 b	1.9 a	4.8 b	8.4 b	
Liao and Burns, 2012^II^	April 2009	Healthy	–	–	–	7.1 a	–	–
		HLB-AS				6.8 a	
		HLB-SY				1.8 b	
Slisz et al., 2012^III^	May 2007	Healthy	1.4 a	1.7 a	4.1 a	–	0.64 a	0.26 a
		HLB-AS	1.3 a	1.6 a	3.7 a		0.57 a	0.23 a
	June 2007	Healthy	1.4 a	1.7 a	4.6 a		0.47 a	0.26 a
		HLB-AS	1.2 a	1.6 a	3.9 a		0.38 a	0.22 a
		HLB-SY	1.1 a	1.5 a	1.5 b**		0.91 b*	0.18 b*
Raithore et al., 2015^IV^	April 2009	Healthy	2.2 a	2.3 a	5.0 a	–	0.53 b	0.17 a
		HLB-SY	2.7 a	2.7 a	3.4 b		1.40 a	0.12 b
Baldwin et al., 2018^V^	April 2015	Healthy	2.0	2.2	5.4	9.6	0.74	0.20
		HLB-SY	2.2	2.5	5.2	9.8	0.80	0.19
Dala Paula et al., 2018^VI^	March 2013	Healthy	2.0 b	2.3 b	5.6 a	10.0 a	0.84 b	0.14 a
		HLB-SY	2.3 a	2.7 a	4.2 b	9.0 b	1.41 a	0.11 b
**HAMLIN ORANGE JUICE**								
Baldwin et al., 2010^I^	December 2007	Healthy	1.5 a	1.5 a	3.9 a	7.0 a	–	–
		HLB-AS	1.3 b	1.4 a	3.2 b	6.0 b	
	February 2008	Healthy	2.2 a	2.2 a	5.4 a	9.8 a	
		HLB-AS	1.8 b	1.8 b	4.0 b	7.6 b	
Raithore et al., 2015^IV^	January 2009	Healthy	2.9 a	3.0 a	5.4 a	–	0.53 a	0.16 a
		HLB-SY	2.7 a	2.7 a	4.7 a		0.55 a	0.17 a
Baldwin et al., 2018^V^	December 2014	Healthy	1.6	1.6	4.6	7.8	0.63	0.21
		HLB-SY	1.7	1.8	3.8	7.3	0.65	0.23
	January 2015	Healthy	2.2	2.2	5.5	9.9	0.66	0.23
		HLB-SY	2.4	2.4	5.3	10.0	0.73	0.21

*FJ, filtered juice; JWP, juice with pulp; AS, asymptomatic; SY, symptomatic; Healthy-R, fruit harvest from healthy not shaken trees; Healthy-R, fruit harvest from healthy shaken trees (healthy-retain); Healthy-D, healthy fruit that dropped to the ground upon shaking the trees (healthy-drop); HLB-R, fruit retained on shaken HLB affected-trees; HLB-D, fruit that dropped from HLB affected-trees*.

Values from the same reference with the same letter within columns and same harvest time do not differ in disease status, according to statistical analysis (^I^Fisher's test significant difference test at p ≤ 0.05; ^II^Duncan's multiple range test p ≤ 0.01; ^III^p-values represent comparisons within harvest *p ≤ 0.05; **p ≤ 0.001; ^IV^ANOVA and Tukey's test p ≤ 0.05; ^V^not applicable; ^VI^ANOVA and Tukey's test p ≤ 0.05 for glucose, total sugars and malic acid, and p ≤ 0.01 for sucrose, fructose and citric acid).

For organic acids, the majority of the studies reported similar citric and ascorbic acid levels in juice from HLB-unaffected fruit and asymptomatic oranges from HLB-affected trees. However, juice from HLB-SY oranges generally has higher content of citric acid and lower content of malic acid compared to juice from healthy fruit (Table 4). Poiroux-Gonord et al. (2013) reported an increase in organic acid, especially succinic acid, in the pulp of oranges nearby leaves submitted to photooxidative stress, a situation associated with HLB effects in citrus leaves (Cen et al., 2017).

#### Secondary Metabolites

Oranges are an important source of secondary metabolites which promote human health, particularly flavonoids, limonoids, hydroxycinnamic acids, and polyamines. Many secondary metabolites result from the interaction between the plant and its environment, and are induced by biotic and abiotic factors. Changes in the levels of certain classes of secondary metabolites in oranges are frequently due to stress conditions in plants, including the photooxidative stress in nearby leaves (Poiroux-Gonord et al., 2013). In addition, these compounds are influenced by many factors, such as: cultivar, cultivating methods, degree of ripeness, and processing and storage conditions (Sudha and Ravishankar, 2002; Ramakrishna and Ravishankar, 2011; Chin et al., 2014).

Generally, higher concentrations of phenolic compounds are found in sprouts and seedlings compared to mature plants, consistent with the notion that plant phenolics provide a degree of protection against predation (Drewnowski and Gomez-Carneros, 2000). Similarly, there is an increase of phenolic compounds levels in fruit and leaves from HLB-infected trees (Dagulo et al., 2010; Hijaz et al., 2013a; Kiefl et al., 2018). Flavonoids, particularly hesperidin, narirutin and dydimin, were higher in the peel, pulp and juice of HLB-symptomatic fruit (Massenti et al., 2016; Dala Paula et al., 2018; Kiefl et al., 2018) in comparison with the respective fruit parts from unaffected trees. The pulp of HLB-symptomatic Valencia oranges from two different harvests (March and May 2013) showed an increase of 148 and 17% in narirutin, respectively, and an increase of 86 and 94% in hesperidin, respectively, compared to the corresponding healthy fruit pulp (Massenti et al., 2016). Juice from HLB-SY Valencia oranges harvested in March 2013, contained higher amounts of tangeretin (>4x), nobiletin (>2x), heptamethoxyflavone (>1.5x), diosmin (>2x), didymin (>1.5x), vicenin-2 (>1.5x), nomilin (>20x), limonin (>7.5x), and limonin glucoside (>1.5x) compared to juice from HLB-unaffected oranges (Dala Paula et al., 2018).

In general, juice made with asymptomatic oranges from HLB-infected trees is more similar to juice made with oranges from HLB-unaffected trees when compared to juice made with symptomatic fruit regarding secondary metabolite content. When differences are present, they are caused by harvest maturity rather than by disease status (Baldwin et al., 2010). The interaction of fruit maturity and HLB is not well understood, but Dagulo et al. (2010) suggested that fruit symptomatic for HLB are similar to immature fruit (lower sugars, higher acids, higher bitter limonoids), which is probably why the effect of HLB is more prevalent early in the season. They also suggested that HLB-affect fruit are slow to mature, likely due to a compromised vascular system. Baldwin et al. (2010) determined several secondary metabolites, including hydroxycinnamic acids at 6.3 min and 7.2 min; vicenin-2; feruloyl putrescine; narirutin 4′-glucoside; limonin glucoside; narirutin; nomilin glucoside; nomilinic acid glucoside; limonin and nomilin in asymptomatic and healthy juice made with Hamlin oranges harvested in December 2007. Feruloyl putrescine was the only secondary metabolite that was present at similar levels. However, the same orange cultivar harvested in February 2008 presented similar levels of the two hydroxycinnamic acids; vicenin-2; feruloyl putrescine, limonin glucoside, narirutin, and nomilin glucoside between healthy and asymptomatic juices. The same comparison performed with Valencia oranges harvested in April 2008, had similar contents of all of the secondary metabolites; however, oranges from the June harvest showed different levels of feruloyl putrescine, limonin glucoside, and limonin. These results demonstrate that harvest maturity has greater effect on the content of secondary metabolites than *C*Las infection (Baldwin et al., 2010).

Juice made with HLB-affected oranges contains high levels of nomilin and limonin, more so when made from symptomatic oranges. Both, nomilin and limonin are known to provide bitterness in citrus fruit and its juice (Maier et al., 1977, 1980; Hasegawa et al., 2000). Early research on the effect of HLB on fruit quality suggested that limonin levels >1 mg/L could induce bitterness in juice (Plotto et al., 2010) as it was also the detection threshold in water (Guadagni et al., 1973). However, further research showed that the recognition threshold of limonin was actually around 4–6 mg/L in a complex matrix such as orange juice (Guadagni et al., 1973; Dea et al., 2013). In fact, it is now recognized that only symptomatic oranges have their taste compromised (Baldwin et al., 2010; Plotto et al., 2010; Slisz et al., 2012; Chin et al., 2014; Raithore et al., 2015; Dala Paula et al., 2018) and only severely affected orange juice has limonin levels above 4 mg/L (Table 5). This suggests that there are other compounds involved with the bitter taste of juice from symptomatic oranges (Dala Paula et al., 2018), and that interactions of flavonoids together with the combination of lower sugars with higher acids enhances limonoid bitterness perception (Dea et al., 2013; Kiefl et al., 2018).

**Table 5:** Limonin-glucoside, limonin and nomilin contents of Valencia and Hamlin orange juice made with healthy fruit and fruit at different stages of HLB infection.

**References**	**Orange juice sample**		**Secondary metabolites (mg/L)**		
	**Harvest time**	**Status or conditions**	**Limonin-glucoside**	**Limonin**	**Nomilin**
**VALENCIA ORANGE JUICE**					
Baldwin et al., 2010^I^	March 2007	Healthy	123.2 a	0.90 b	0.22 b
		HLB-AS	123.4 a	1.37 a	0.66 a
	April 2007	Healthy	122.4 b	0.78 b	0.30 b
		HLB-AS	137.6 a	1.24 a	0.54 a
	May 2007	Healthy	134.9 a	0.67 b	0.12 b
		HLB-AS	137.7 a	1.40 a	0.26 a
	June 2007	Healthy	115.4 b	0.52 b	0.06 a
		HLB-AS	144.4 a	0.93 a	0.11 a
Slisz et al., 2012^II^	May 2007	Healthy	530 b	3.29 b	–
		HLB-AS	716 a***	4.71 a*
	June 2007	Healthy	716 a	2.82 c
		HLB-AS	911 a	5.18 b**
		HLB-SY	976 a	7.53 a
Raithore et al., 2015^III^	April 2009	Healthy	–	0.85 b	0.22 b
		HLB-SY		2.34 a	0.69 a
Kiefl et al., 2018^IV^	February 2015	Healthy	240	10	<LOQ***
		HLB-SY	>250	11	<LOQ
	March 2015	Healthy	180	<LOQ**	nd
		HLB-SY	>250	8	<LOQ
	April 2015	Healthy	220	<LOQ	nd
		HLB-SY	>250	<LOQ	nd
Baldwin et al., 2018^IV^	April 2015	Healthy	147.8	1.4	0.1
		HLB-SY	126.9	4.2	1.4
Dala Paula et al., 2018^V^	March 2013	Healthy	48.3 b	1.2 b	0.1 b
		HLB-SY	92.0 a	9.3 a	1.1 a
**HAMLIN ORANGE JUICE**					
Baldwin et al., 2010^I^	December 2007	Healthy	72.3 b	1.45 b	0.43 b
		HLB-AS	102.0 a	3.27 a	0.83 a
	February 2008	Healthy	132.1 a	0.82 b	0.18 b
		HLB-AS	141.7 a	1.54 a	0.51 a
Raithore et al., 2015^III^	January 2009	Healthy	–	0.64 b	0.06 b
		HLB-SY		2.44 a	0.25 a
Kiefl et al., 2018^IV^*	November 2014	Healthy	110	8.3	9.7
		HLB-SY	>250	16	11
	January 2015	Healthy	140	<LOQ	<LOQ
		HLB-SY	>250	13	<LOQ
Baldwin et al., 2018^IV^	December 2014	Healthy	33.7	1.3	0.1
		HLB-SY	34.9	2.2	0.3
	January 2015	Healthy	47.2	0.8	0.1
		HLB-SY	56.1	1.1	0.2

*< LOQ, below limit of quantification; nd, not detectable*.

Values from the same reference with the same letter within columns and same harvest time do not differ in disease status, according to statistical analysis (^I^Fisher's test significant difference test at p ≤ 0.05 for limonin glucoside and p ≤ 0.01 for limonin and nomilin; ^II^p-values represent comparisons within harvest *p ≤ 0.05; **p ≤ 0.001; ^III^ANOVA and Tukey's test p ≤ 0.05; ^IV^not applicable; ^V^ANOVA and Tukey's test p ≤ 0.001).

* *The results were converted from mg/Kg to mg/L assuming orange juice's density of 1.0 g/cm^3^*;

** *LOQ of limonin = 1.2 mg/Kg*;

*** *LOQ of nomilin = 5.0 mg/Kg*.

#### Amino Acids and Bioactive Amines

The accumulation of proline, arginine, and branched chain amino acids is expected in plants subjected to conditions that induce stress, such as drought, high salinity and acidity, high incidence of light, high concentration of heavy metals in the soil, changes in temperature, as well as response to biotic stress, such as plant diseases (Rai, 2002; Sharma and Dietz, 2006; Slisz et al., 2012; Malik et al., 2013). Studies showed that proline was higher in leaves of symptomatic HLB-infected trees (Cevallos-Cevallos et al., 2011, 2012; Malik et al., 2014), but it was lower in juice from HLB-SY Valencia fruit (Slisz et al., 2012). In contrast, Hung and Wang (2018) reported an accumulation of proline in Hamlin orange juice from HLB-infected trees. These authors suggested that some of the control trees of the Slisz et al. (2012) study possibly tested as false negatives due to the detection limit of PCR methods or uneven distribution of *C*Las throughout the tree. However, in both studies the amino acids alanine, arginine, leucine, isoleucine, threonine, and valine were found at lower concentrations in juice from HLB-symptomatic oranges (Slisz et al., 2012; Hung and Wang, 2018).

In juice from HLB-symptomatic Valencia and Hamlin oranges, the concentrations of asparagine and phenylalanine were over two times higher than in juice from healthy oranges, and histidine content also increased (Chin et al., 2014). An increase of asparagine and histidine contents was also found in juice from HLB-symptomatic Valencia fruit (Slisz et al., 2012) and in Satsuma orange leaves (Malik et al., 2014). A suggested explanation for this trend is that *C*Las may have inhibited the tree defense mechanism which, in turn, reduced the action of proline dehydrogenase, an enzyme responsible for the activation of the biosynthetic pathways of proline from ornithine and glutamate. Thus, the levels of this amino acid could not increase (Slisz et al., 2012). However, the accumulation of phenylalanine in juice from HLB-affected oranges (Slisz et al., 2012) differs from results from Malik et al. (2014) and Hung and Wang (2018). These last authors explained that phenylalanine is an essential precursor for secondary phenylpropanoid metabolism by phenylalanine ammonialyase in higher plants and its gene expression is significantly affected by *C*Las infection (Hung and Wang, 2018).

Hamlin and Valencia HLB-symptomatic oranges showed high contents of the aromatic amine synephrine, however, juice from HLB-asymptomatic and healthy fruit had similar content (Slisz et al., 2012; Chin et al., 2014). In plants, putrescine is a necessary diamine precursor of polyamines synthesis (spermidine and spermine), and its increase is usually associated with environmental stress (Coelho et al., 2005; Gloria, 2006; Sharma and Dietz, 2006); however, putrescine content was not affected in juice from HLB-symptomatic oranges (Chin et al., 2014). On the other hand, feruloyl putrescine, a conjugate of putrescine and ferulic acid, is found at high concentrations in juice from HLB-symptomatic Hamlin oranges compared to juice from HLB-asymptomatic and healthy fruit. The same trend does not seem to be observed in Valencia oranges (Baldwin et al., 2010).

### Effect of HLB on the Levels and Profile of Volatile Compounds

The orange flavor has been studied more than any other citrus flavor. Unlike grapefruit, lemon, and lime, in which there are one or two flavor-impact compounds, the orange flavor is the result of a combination of volatiles in specific proportions. Among the various components that contribute to the distinct flavor of the orange, the most important are: terpenes (d-limonene, myrcene, α-pinene, valencene); aldehydes (acetaldehyde, *E*-2-pentenal, hexanal, octanal, nonanal, decanal, sinensal, neral, and geranial, the last two sometimes called citral); esters (ethyl acetate, ethyl propionate, methyl butanoate, ethyl butanoate, ethyl 2-methylpropanonate, ethyl 2-methylbutanoate, ethyl 3-hydroxyhexanoate); alcohols (ethanol, *E*-2-hexen-1-ol, *Z*-3-hexen-1-ol, linalool, α-terpineol); and ketones (1-octen-3-one, β-damascenone, β-ionone) (Shaw, 1991; Perez-Cacho and Rouseff, 2008).

**Figure 3:**
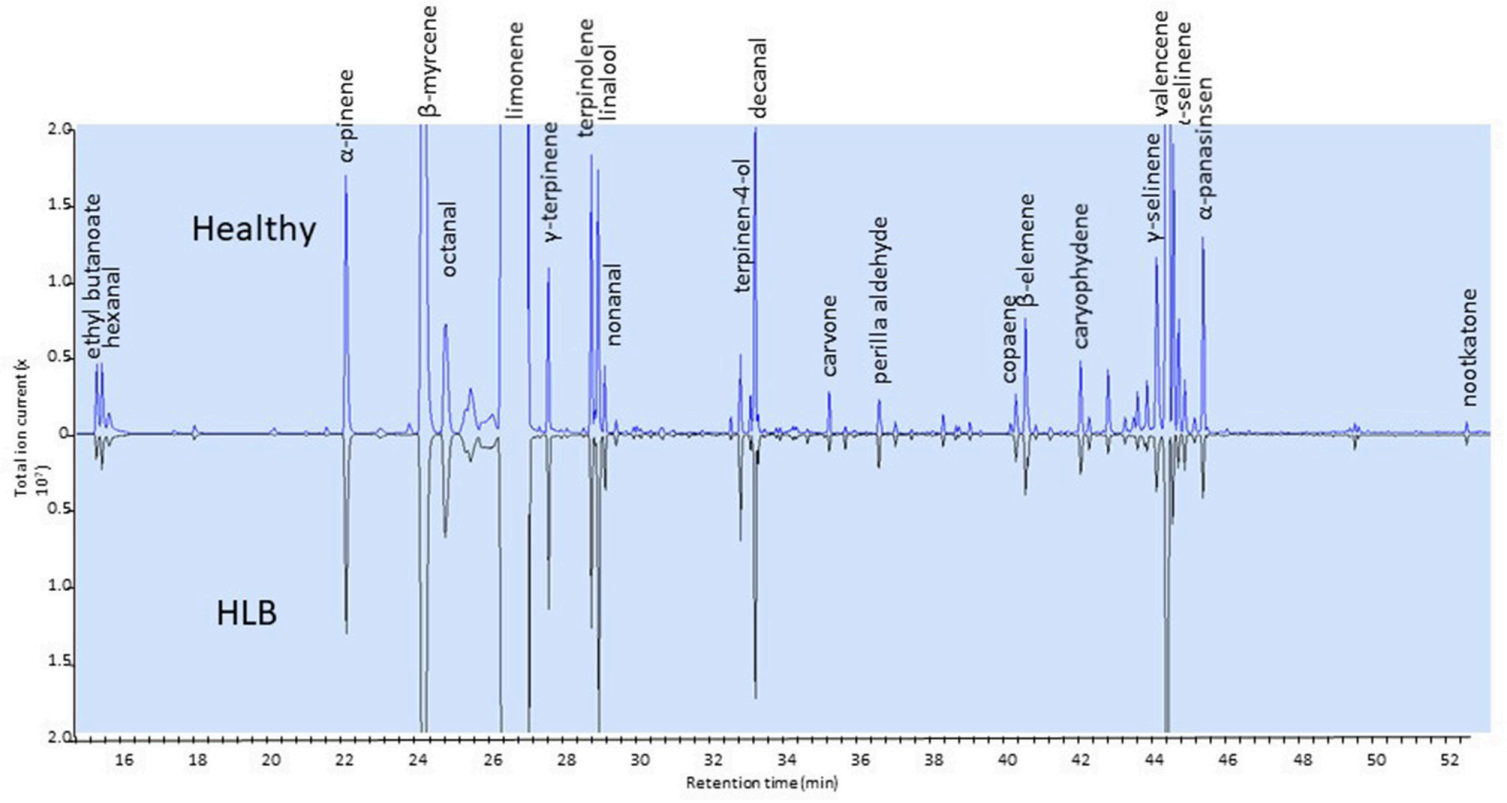
Side-by-side chromatograms of headspace volatiles of juice samples extracted from healthy **(top)** and HLB **(bottom)** Valencia oranges. Ethyl butanoate (ester) and sesquiterpenes are in greater amount in healthy than in HLB juice.

Only a few studies have dealt with changes in the volatile compounds in orange juice affected by HLB (Baldwin et al., 2010; Dagulo et al., 2010; Hung and Wang, 2018; Kiefl et al., 2018). These studies have shown that monoterpenes tend to be higher and esters lower in juice affected by HLB (Baldwin et al., 2010; Dagulo et al., 2010; Kiefl et al., 2018). These studies have also shown that sesquiterpenes, including valencene, were typically lower in HLB-affected juice (Figure 3). These results are relevant to the quality of orange juice as esters typically impart fruity flavor and terpenes are characteristic of citrus volatiles: ethyl acetate, ethyl butanoate and ethyl hexanoate have sweet fruity odors in orange juice (Plotto et al., 2008). Ethyl-3-hydroxyhexanoate is reported as one of the major esters in orange juice (Shaw, 1991; Fan et al., 2009) with a sweet and fruity odor (Buettner and Schieberle, 2001). Lower esters and higher terpenes are likely to result in imbalanced flavor of orange juice. While the terpene alcohol linalool, with a fruity/floral characteristic, is desired in orange juice, other terpene alcohols (α-terpineol, 4-terpineol, carveol) are indicators of oxidation and poor quality (Dagulo et al., 2010; Kiefl et al., 2018). Dagulo et al. (2010) suggested that the higher terpenes and lower sesquiterpenes in HLB-affected orange juice might be an indication of lower enzyme activity in the pathway converting terpenes to sesquiterpenes of the affected oranges.

Contradictory results were reported for alcohols. Dagulo et al. (2010) and Baldwin et al. (2010) found that (*Z*)-3-hexenol was higher in juice from HLB-affected Valencia oranges, while Kiefl et al. (2018) found it was higher in juice from healthy fruit. In fact, Dagulo et al. (2010) and Hung and Wang (2018) found that all alcohols were higher in HLB-affected juice. The levels of aldehydes varied much more depending on the study, season and cultivar. Octanal, nonanal, and decanal are important aldehydes with a characteristic citrus odor (Perez-Cacho and Rouseff, 2008) and were higher in juice from “healthy” oranges in the Kiefl et al. (2018) and Baldwin et al. (2010) studies. On the contrary, these aldehydes were higher in juice from HLB-asymptomatic Valencia oranges in the Dagulo et al. (2010) study. Likewise, the “green” odor compound hexanal was 65 to 110% higher in samples from HLB-unaffected samples in the Baldwin et al. (2010) study, up to 81% higher in HLB-symptomatic Valencia in the Dagulo et al. (2010) study and about 25% higher in HLB-affected fruit (Kiefl et al., 2018). Considering all three studies, it is important to remember that volatile levels differ with harvest times, types of processes used to prepare the orange juice (Baldwin et al., 2012b) and HLB infection status. It is important to emphasize that, generally, asymptomatic orange juice is similar to healthy orange juice with respect to volatile profile.

Not only does HLB affect the profile of volatiles in orange juice, but by having an effect on fruit size, peel oil extracts are reduced by 30% in HLB-symptomatic fruit (Bai et al., 2017). As in orange juice, sesquiterpene hydrocarbons are lower in the peel oil of symptomatic fruit, as are some monoterpenes and straight-chain aldehydes. In another study, Xu et al. (2017) found compounds only detected in oil from HLB-affected fruit, including β-longifolene and perillene, two terpenes, and 4-decenal, an aldehyde. However, these authors admit that more samples should be analyzed to confirm these findings. These authors found that linalool, decanal, citronellol, citral, carvone, and dodecanal were higher in the oil from asymptomatic than symptomatic fruit from Hamlin and Valencia oranges harvested twice in the season (Xu et al., 2017). Kiefl et al. (2018) analyzed peel oil by gas chromatography and olfactometry and found that mostly odor-active aldehydes contributed to the difference between healthy and HLB-affected Valencia oil, being higher in HLB-affected fruit.

Emission of volatiles from orange tree is believed to play an important role in the plant—vector APC interaction. Orange leaves emit almost all juice volatiles except esters (Hijaz et al., 2013b, 2016). ACP infestation stimulated 21 out of the 27 volatiles by 2- to 10-fold in orange leaves, in comparison with *C*Las-infection which only stimulated four volatiles—d-limonene, β-phelandrene, citronellal, and undecanal by 4- to 20- fold (Hijaz et al., 2013b). In another experiment, Hijaz et al. (2016) showed that HLB tolerant cultivars contained higher amounts of volatiles, especially those showing antimicrobial activities, including aldehydes (undecanal, neral, geranial, and citronellal), and mono/sesquiterpene hydrocarbons and derives (linalool, d-limonene, myrcene, α- and β- phellandrene, E-caryophellene, β- and γ-elemene, germacrene D, and geranyl acetate).

### Effects of HLB on Juice Sensory Characteristics

Early reports describing the symptoms of HLB disease on trees, leaves, and citrus fruit were published in plant pathology journals, and effects on fruit were mostly describing the visual defects. One report mentioned HLB-symptomatic oranges as having a “bitter and salty taste, especially in the early part of the season” (McClean and Schwarz, 1970). These were informal observations about fruit having off flavor. Only recently formal sensory analyses (triangle test, difference-from-control test) have been used to describe and quantify other, and more subtle taste attributes in HLB-affected fruit (Plotto et al., 2008). Studies have included analysis of juice prepared from fruit of healthy, unaffected trees, and of juice prepared from asymptomatic and symptomatic fruit from HLB-affected trees testing positive for *C*Las. Comparisons were made using difference-from-control tests where panelists rated the degree of difference between healthy and infected juice. Sensory results could be explained by chemical data and confirmed differences between healthy, asymptomatic, and symptomatic fruit/juice. These comparisons were repeated with several cultivars, Hamlin, Mid-Sweet and Valencia, and the differences between healthy and HLB-affected fruit were more pronounced and obvious to the palate with fruit harvested early than late in the season (Baldwin et al., 2010; Plotto et al., 2010). Juice made with these symptomatic, HLB-affected oranges had the most off-flavors, commonly described as “bitter,” “sour,” and “sour/fermented.” Higher bitterness and sourness in symptomatic fruit could be explained by higher levels of limonin and titratable acidity and with lower soluble solids content (Baldwin et al., 2010). A trained panel provided more insight into the various descriptors characterizing orange juice made with HLB-symptomatic fruit, with several negative descriptors regarding taste and flavor (astringency, tingling, harshness, bitterness, metallic-taste, low sweetness, saltiness/umami, musty, sourness/fermented, pungent/peppery, low citrusy taste; Tables 6, 7), usually due to an imbalance in the chemical composition in the affected fruit (Baldwin et al., 2010, 2012a, 2018; Plotto et al., 2010, 2017; Raithore et al., 2015; Dala Paula et al., 2018; Kiefl et al., 2018).

**Table 6:** Sensorial descriptors ascribed to Huanglongbing in Valencia orange juice.

Sensorial descriptor*	**Harvest time**	**Juice specifications**	**References^#^**
Acidic	July 2006, April 2008	Frozen juice with pulp and filtered^I^, hand-squeezed juice ^II^	1; 4
Astringent	June 2008; March 2013; April 2015	Commercially processed juice^III^; premium setting^IV^	2; 5; 6
Bitter/slight bitter	June 2008; March 2013	Commercially processed juice	2; 6
Bland	June 2008	Commercially processed juice	2
Burning	March 2013; April 2015	Premium setting, commercially processed juice	5; 6
Fermented	July 2006	Frozen juice with pulp and filtered	1
Grapefruit-like flavor	April 2008, 2015; June 2008; March 2013;	Commercially processed juice, premium setting	2; 5; 6
Green flavor	March 2013; April 2015	Premium setting, commercially processed juice	5; 6
HLB-bitter***	Monthly basis during the season 2014 and 2015	Hand-squeezed juice	4
Less body**	April 2015	Premium setting	5
Less fruity non-citrus flavor**	March 2013; April 2015	Premium setting, commercially processed juice	5; 6
Less orange flavor**	March 2013; April 2015	Premium setting, commercially processed juice	5; 6
Less sweet**	April 2008, 2015; March 2013	Commercially processed juice, premium setting	2; 5; 6
Metallic	June 2008; April 2009; 2015	Commercially processed juice	3; 5
Off flavor	April 2008	Commercially processed juice	2
Overripe	July 2006	Frozen juice with pulp and filtered	1
Oxidized oil	April 2015	Premium setting	5
Peel oil	April 2008, 2015; June 2008; March 2013;	Commercially processed juice, premium setting	2; 5; 6
Salty/umami	April 2009; March 2013	Commercially processed juice	3; 6
Sharp	April 2008, June 2008	Commercially processed juice	2
Sour	April 2008; 2009; 2015; Mar 2013;	Commercially processed juice, premium setting	2; 3; 5; 6
Stale	March 2013	Commercially processed juice	6
Sweeter**	April 2008	Commercially processed juice	2
Tangy	April 2008	Commercially processed juice	2
Tingly	April 2009; 2015	Commercially processed juice, premium setting	3; 5
Typical HLB flavor	March 2013; April 2015	Premium setting, commercially processed juice	5; 6
Umami	April 2015	Premium setting	5
Unidentifiable different flavor	June 2008	Commercially processed juice	2
Weak in taste	July 2006	Frozen juice with pulp and filtered	1

* *The list of sensorial descriptors includes commentaries realized by the panel during sensory evaluations and attributes significantly higher in asymptomatic or symptomatic orange juice, CLas (+), compared to healthy juice (control)*.

** *In comparison with healthy orange juice (control), CLas (–)*.

*** According to the authors, HLB-bitter refers to a long-lasting metallic, astringent and harsh taste.

I Frozen juice thawed overnight served with the pulp and without pulp. Juice was filtered then flash pasteurized at 71°C for 10 s and immediately cooled then served;

II Oranges were hand juiced and lightly pasteurized using at 71°C for 15 s, and frozen at −20°C;

III *Oranges were extracted using a commercial JBT 391 single head extractor with premium juice extractor settings and pasteurized under simulated commercial conditions (1.2 L/m, 8 to 10 s hold time, 83 to 90°C)*.

IV *Oranges were extracted as is a customary industry practice, premium setting was selected according to the particular characteristic of the peel oil specific to Valencia, it was passed through a pressure filtration finisher with screen size 0.51 mm and then pasteurized under simulated commercial conditions (1.2 L/m, 90°C)*.

# *References: ^1^Plotto et al. (2008); ^2^Plotto et al. (2010); ^3^Raithore et al. (2015); ^4^Kiefl et al. (2017); ^5^Baldwin et al. (2018); ^6^Dala Paula et al. (2018)*.

**Table 7:** Sensorial descriptors ascribed to Huanglongbing in Hamlin orange juice.

Sensorial descriptor*	**Harvest time**	**Juice specifications**	**References^#^**
Astringent	February 2008; December 2014; January 2015	Commercially processed juice^I^, standard industry setting^II^	1; 4
Bitter	December 2007; 2014; February 2008; January 2009; 2015	Hand-squeezed juice^III^, commercially processed juice, standard industry setting	1; 2; 4
Burning	December 2014; January 2015	Standard industry setting	4
Cooked	January 2009	Commercially processed juice	2
Earthy	February 2008	Commercially processed juice	1
Fatty	February 2008	Commercially processed juice	1
Fermented	February 2008	Commercially processed juice	1
Grapefruit-like	December 2007; 2014; February 2008; January 2009; 2015	Hand-squeezed juice, commercially processed juice, standard industry setting	1; 2; 4
Green flavor	December 2014; January 2015	Standard industry setting	4
HLB bitter***	Monthly basis during the season 2014 and 2015	Hand-squeezed juice	3
Less body**	December 2014	Standard industry setting	4
Less freshness**	February 2008	Commercially processed juice	1
Less fruity non-citrus flavor**	December 2014	Commercially processed juice	4
Less orange flavor**	February 2008	Commercially processed juice	1
Less sweet**	February 2008; December 2014; January 2015	Commercially processed juice, standard industry setting	1, 4
Metallic	February 2008; December 2014; January 2015	Commercially processed juice, standard industry setting	1; 4
Musty	February 2008	Commercially processed juice	1
Overripe	January 2009	Commercially processed juice	2
Oxidized oil	December 2014; January 2015	Standard industry setting	4
Peel oil/citrus oil	December 2007; 2014; February 2008; January 2009; 2015	Hand-squeezed juice, commercially processed juice, standard industry setting	1; 2; 4
Peppery	February 2008	Commercially processed juice	1
Pungent	February 2008	Commercially processed juice	1
Salty/umami	February 2008	Commercially processed juice	1
Sharp	December 2007	Hand-squeezed juice	1
Sour	December 2007; 2014; February 2008; January 2009; 2015	Hand-squeezed juice, commercially processed juice, standard industry setting	1; 2; 4
Stale	December 2014; January 2015	Standard industry setting	4
Sour milk	December 2007	Hand-squeezed juice	1
Sulfury	January 2009	Commercially processed juice	2
Tingly	February 2008; December 2014; January 2015	Commercially processed juice, standard industry setting	1; 4
Typical HLB flavor	December 2014; January 2015	Standard industry setting	4
Umami	December 2015; January 2015	Standard industry setting	4

**#x0002A;:** The list of sensorial descriptors includes commentaries realized by the panel during sensory evaluations and attributes significantly higher in asymptomatic or symptomatic orange juice, CLas (+), compared to healthy juice (control);

** *In comparison with control juice—healthy orange juice, CLas (–)*.

*** According to the authors, HLB-bitter refers to a long-lasting metallic, astringent and harsh taste.

I *Oranges were extracted using a commercial JBT 291 single head extractor with premium juice extractor settings and pasteurized under simulated commercial conditions (1.2 L/m, 8 to 10 s hold time, 82 to 90°C)*.

II Oranges were extracted as is a customary industry practice, premium setting was selected according to the particular characteristic of the peel oil specific to Hamlin, it was passed through a pressure filtration finisher with screen size 0.51 mm and then pasteurized under simulated commercial conditions (1.2 L/m, 90°C).

III Oranges were hand juiced and lightly pasteurized using at 71°C for 15 s, and frozen at −20°C;

# *References: ^1^Plotto et al. (2010); ^2^Raithore et al. (2015); ^3^Kiefl et al. (2017); ^4^Baldwin et al. (2018)*.

HLB off-flavor in severely symptomatic fruit is so pronounced that processing healthy with affected fruit is likely to negatively impact the sensory quality of commercial orange juice (Bassanezi et al., 2009). Juice from HLB-symptomatic fruit, up to 25%, can be blended with juice from unaffected fruit without being perceived as off-flavored for both Hamlin and Valencia (Raithore et al., 2015). Another study found an even lower amount (10% by juice mass) of HLB-symptomatic fruit being acceptable in a blend (Ikpechukwu, 2012). Both studies were performed with not-from-concentrate juice processed in a pilot plant, and can be a basis to processors who need to sort symptomatic fruit out before juicing to maintain overall juice quality (Raithore et al., 2015). No studies were found with juice made from concentrate, but processors always blend those juices and add volatiles which can mask some off-flavors.

More in-depth studies on bitterness in orange juice revealed that the two known bitter limonoids in orange juice, limonin, and nomilin, act in a synergistic manner and their thresholds of perception are lower when tasted together (Dea et al., 2013). Furthermore, both limonoids have a different taste characteristic: limonin is described as “bitter” whereas nomilin is described as “metallic” by some panelists, probably contributing to the taste synergy. Unlike other tastes, the detection thresholds for bitter molecules are generally extremely low, and can have prolonged aftertaste. Perception of bitterness is highly variable among humans, and because there are more than 50 known bitter receptors, studies of bitterness associated with juice affected by HLB are complex. Fractionated liquid chromatography of orange juice combined with taste analysis revealed that derivative molecules of hydroxycinnamic acids had bitter and astringent taste, and were more prevalent in juice from HLB-symptomatic oranges (Dala Paula et al., 2018). Using the same technique, Glabasnia et al. (2018) identified 10 polymethoxylated flavones (PMFs) that enhanced bitterness due to limonin and nomilin in orange juice. Tasted without limonin and nomilin in a model solution, these PMFs increased astringency but not bitterness. These studies demonstrate the complexity of interactions between molecules belonging to two chemical classes—polyphenols and limonoids, on taste perception. Contribution of volatiles, sugars, acids, amino acids, and high molecular weight carbohydrates such as pectin to flavor and taste adds to the complexity of understanding the effect of HLB on juice quality.

A new technology was developed to predict HLB-affected orange juice quality by measuring pathogen *C*Las titer using real-time PCR (Bai et al., 2013; Zhao et al., 2018). Fruit severely infected by HLB may have one or more of the following juice quality features: low sugar, abundant bitter limonoids, and rich acid/sourness, but the common feature for all juice prepared from such fruit is high *C*Las titer, which correlated negatively with sensory characteristics (Bai et al., 2013; Zhao et al., 2018). The U.S. patent by Zhao et al. (2018) is the only study where *C*Las is quantified in orange juice from many sources showing an attempt of quantifying the degree of infection. The amount of *C*Las titer in the juice (lower CT values) negatively correlated with sweetness, orange and fruity flavor, and positively with negative attributes, such as off flavor and “umami.”

## Final Considerations

HLB affects the sensory and physicochemical characteristics of orange juice despite the available scientific literature data which presents contradictory results among these parameters. This may be due to factors such as: different harvest times of the oranges, differences in the age of the trees between the control group and HLB group, unpredictable environmental stress, as well as the level of *C*Las infection of the orange trees. Juice made with HLB-symptomatic fruit usually has high TA, low SSC and SSC/TA, whereas juice made with asymptomatic fruit from HLB-infected trees is generally similar to juice processed with healthy fruit. In general, HLB causes a decrease in sucrose, total sugars and malic acid contents while ascorbic acid does not seem to be significantly affected by the disease. On the other hand, levels of citric acid, bitter limonoids (limonin and nomilin), hydroxycinnamic acids, flavonoids (particularly tangeretin), nobiletin, narirutin, hesperidin, diosmin, and didymin are higher in juice from HLB-symptomatic oranges compared to juice from healthy fruit. The content of amino acids, alanine, arginine, asparagine, histidine, isoleucine, leucine, phenylalanine, proline, threonine, and valine are altered by HLB. Additionally, symptomatic Hamlin orange juice has high synephrine and feruloyl putrescine levels.

Regarding the typical HLB-off flavor in orange juice, the loss of sweetness can generally be explained by lower sucrose and total sugar levels and SSC, along with higher citric acid, and sourness is explained by higher TA and citric acid content. Furthermore, some volatiles may contribute to increased or decreased perception of sweetness or sourness (Bartoshuk et al., 2017; Plotto et al., 2017). Elevated levels of limonin and nomilin are partially responsible for the typical HLB-bitterness. These two limonoids have a synergistic effect which decreases their perception and identification thresholds in orange juice. Beyond these compounds, there is evidence indicating that other compounds, possibly hydroxycinnamic acids, are involved with the typical HLB-bitterness (Dea et al., 2013; Dala Paula et al., 2018). Unquestionably, more work is needed to further identify the full list of compounds contributing to the unpleasant taste and mouthfeel in HLB-affected orange juice. Sensory studies take into consideration that the lower sugar contents reinforce the perception of bitterness.

There are relatively few published papers evaluating the effects of HLB on orange juice's chemical, physicochemical and, especially, sensorial qualities and most of the research available was performed using Valencia oranges, followed by Hamlin. While citrus fruit sold as fresh can be substantially devalued by loss of color and misshape, juice processors still can process oranges that are HLB-symptomatic as long as they are mixed with asymptomatic fruit in < 25% ratio of HLB-SY to asymptomatic fruit (healthy or HLB-AS). Processors traditionally add back flavor extracts from orange peel oil or orange essence to standardize juice (Ringblom, 2004), and have that tool to modulate citrus flavor and sweetness. Other attempts have been made to isolate compounds, or groups of compounds from citrus juice, peel or molasses, which could also increase sweetness or decrease bitterness perception in HLB-affected orange juice (Kiefl et al., 2017). More research to mitigate HLB-induced off-flavors and tastes could include use of resins, that are already used to remove bitter limonoids; the proper resin that only removes bitter compounds without removing flavor volatiles would need to be designed. Also tailoring aroma packages to mask bitterness or enhance sweetness, or adding non-volatiles extracted from oranges that mask bitterness. Finally, perhaps adding substances that bind bitter limonoids in the juice and then remove, or adding enzymes that glycosylate bitter limonoids, rendering them non-bitter. These efforts are likely to be pursued until a long-term solution is found to citrus greening disease.

## Author Contributions

BD-P, AP, JB, JM, EB, RF, and MG contributed to the writing and review of the manuscript.

### Conflict of Interest Statement

The authors declare that the research was conducted in the absence of any commercial or financial relationships that could be construed as a potential conflict of interest. The handling editor is currently co-organizing a Research Topic with one of the authors RF, and confirms the absence of any other collaboration.

## Abbreviations

AS: Asymptomatic
*C*L: *Candidatus* Liberibacter
*C*Laf: *Candidatus* Liberibacter africanus
*C*Lam: *Candidatus* Liberibacter americanus
*C*Las: *Candidatus* Liberibacter asiaticus
DNA: Deoxyribonucleic acid
FJ: Filtered juice
HLB: Huanglongbing
JWP: Juice with pulp
MT: Metric tons
PCR: Polymerase chain reaction
rDNA: Recombinant Deoxyribonucleic acid
SSC: Soluble solids content
SY: Symptomatic
TA: Total acidity.
