# Supplementary material for: Effect of Huanglongbing or Greening Disease on Orange Juice Quality, a Review
Source: Front Plant Sci. 2019 Jan 22;9:1976. doi: 10.3389/fpls.2018.01976 (PMC6350258; doi:10.3389/fpls.2018.01976)
Supplement: Supplementary file 1 [file Table_1.pdf]

Table S. Worldwide distribution of Huanglongbing's bacteria and vectors

Country	Bacteria	Status	Vector	Status
**Asia**				
Afghanistan	-	-	*Diaphorina citri*	present
Bangladesh	*CLas*	present	Diaphorina citri	present
Bhutan	*CLas*	present	*Diaphorina citri*	present
Cambodia	*CLas*	present	*Diaphorina citri*	present
China	*CLas*	present (Fujian, Guangdong, Guangxi, Guizhou, Hainan, Hunan, Jiangxi, Schuan, Yunnan, Zhejiang); few occurrences (Xianggang - Hong Kong)	*Diaphorina citri*	present (Aomen, Fujian, Guangdong, Guizhou, Hainan, Henan, Hunan, Jiangxi, Sichuan, Yunnan, Zhejiang); widespread (Xianggang); restricted distribution (Guangxi)
East Timor	*CLas*	widespread	*Diaphorina citri*	present
India	*CLas*	present (Andhra Pradesh, Arunachal Pradesh, Assam, Bihar, Delhi, Gujarat, Haryana, Himachal Pradesh, Jammu & Kashmir, Karnataka, Kerala, Madhya Pradesh, Maharashtra, Manipur, Meghalaya, Mizoram, Nagaland, Orissa, Punjab, Rajasthan, Sikkim, Tamil Nadu, Tripura, Uttar Pradesh, West Bengal)	*Diaphorina citri*	present (Andhra Pradesh, Arunachal Pradesh, Assam, Bihar, Delhi, Gujarat, Haryana, Himachal Pradesh, Jammu & Kashmir, Karnataka, Kerala, Lakshadweep, Madhya Pradesh, Maharashtra, Manipur, Meghalaya, Punjab, Rajasthan, Sikkim, Tamil Nadu, Tripura, Uttar Pradesh, West Bengal)
Indonesia	*CLas*	present (Irian Jaya, Java, Kalimantan, Sulawesi, Sumatra); widespread (Nusa Tenggara)	*Diaphorina citri*	present (Java, Maluku, Nusa Tenggara, Sumatra);
Iran	*CLas*	restricted distribution	*Diaphorina citri*	restricted distribution
Japan	*CLas*	present (Ryukyu Archipelago); restricted distribution (Kyushu)	*Diaphorina citri*	few occurrence (Kyushu); present (Ryukyu Archipelago)
Laos	*CLas*	present	*Diaphorina citri*	present
Malaysia	*CLas*	present (Sarawak, West)	*Diaphorina citri*	present (Sabah, West)
Maldives	-	-	*Diaphorina citri*	present
Myanmar	*CLas*	present	*Diaphorina citri*	present
Nepal	*CLas*	widespread	*Diaphorina citri*	present
Oman	-	-	*Diaphorina citri*	restricted distribution
Pakistan	*CLas*	present	*Diaphorina citri*	widespread
Philippines	*CLas*	widespread	*Diaphorina citri*	present
Saudi Arabia	*CLaf*	present	*Trioza erytreae*	restricted distribution
			Diaphorina citri	present
Sri Lanka	*CLas*	present	*Diaphorina citri*	present
Taiwan	*CLas*	present widespread	*Diaphorina citri*	restricted distribution
Thailand	*CLas*	present	*Diaphorina citri*	present
United Arab Emirates	-	-	*Diaphorina citri*	present
Vietnam	*CLas*	present restricted distribution	*Diaphorina citri*	restricted distribution
Yemen	*CLaf*	present restricted distribution	*Trioza erytreae*	restricted distribution
			Diaphorina citri	present
**Africa**				
Angola	-	-	*Trioza erytreae*	present
Burundi	*CLaf*	present	-	-
Cameroon	*CLaf*	present	*Trioza erytreae*	present
Camoros	-	-	*Trioza erytreae*	present
Congo, Democratic republic of the	-	-	*Trioza erytreae*	restricted distribution
Central African	*CLaf*	present	-	-

Country	Bacteria	Status	Vector	Status
Republic				
Eritrea	-	-	*Trioza erytreae*	present
Ethiopia	*CLaf*	present	*Trioza erytreae*	present
	CLas	present (few occurrences)		
Kenya	*CLaf*	present	*Trioza erytreae*	present
Madagascar	*CLaf*	present	*Trioza erytreae*	present
Malawi	*CLaf*	present	*Trioza erytreae*	present
Mauritius	*CLaf*	present	*Trioza erytreae*	present
	CLas	restricted distribution	*Diaphorina citri*	present
Réunion	*CLaf*	present	*Trioza erytreae*	present
	CLas	restricted distribution	*Diaphorina citri*	present
Rwanda	*CLaf*	present	*Trioza erytreae*	present
Saint Helena	*CLaf*	present (widespread)	*Trioza erytreae*	present
Sao Tome & Principe	-	-	*Trioza erytreae*	present
Somalia	*CLaf*	present	-	-
South Africa	*CLaf*	restricted distribution	*Trioza erytreae*	widespread
Sudan	-	-	*Trioza erytreae*	present
Swaziland	*CLaf*	present	*Trioza erytreae*	restricted distribution
Tanzania	*CLaf*	restricted distribution	*Trioza erytreae*	restricted distribution
			Diaphorina citri	restricted distribution
Uganda	-	-	*Trioza erytreae*	present
Zambia	-	-	*Trioza erytreae*	present
Zimbabwe	*CLaf*	restricted distribution	*Trioza erytreae*	present
**North America**				
Mexico	*CLas*	restricted distribution	*Diaphorina citri*	restricted distribution
USA	*CLas*	present, few occurrences (California, Georgia, Louisiana, South Carolina, Texas); widespread (Florida)	*Diaphorina citri*	present (Florida, Hawaii, Texas); few occurrences (Alabama, California, Georgia, Louisiana, Mississippi, South Carolina); restricted distribution (Arizona)
**Central America**				
Antigua and Barbuda	-	-	*Diaphorina citri*	present
Bahamas	-	-	*Diaphorina citri*	present
Barbados	*CLas*	restricted distribution	*Diaphorina citri*	restricted distribution
Belize	*CLas*	restricted distribution	*Diaphorina citri*	present
Cayman Islands	-	-	*Diaphorina citri*	present
Costa Rica	*CLas*	restricted distribution	*Diaphorina citri*	present
Cuba	*CLas*	present (widespread)	*Diaphorina citri*	present
Dominica	*CLas*	restricted distribution	*Diaphorina citri*	present
Dominican Republic	*CLas*	restricted distribution	*Diaphorina citri*	present

Country	Bacteria	Status	Vector	Status
Guadeloupe	*CLas*	restricted distribution	*Diaphorina citri*	restricted distribution
Haiti	-	-	*Diaphorina citri*	present
Honduras	*CLas*	present (few occurrences)	-	-
Jamaica	*CLas*	present (widespread)	*Diaphorina citri*	present
Martinique	*CLas*	restricted distribution	*Diaphorina citri*	present
Nicaragua	*CLas*	present	-	-
Puerto Rico	*CLas*	present	*Diaphorina citri*	present
United States Virgin Islands	*CLas*	present (few occurrences)	*Diaphorina citri*	present
**South America**				
Brazil	*CLas*<br>*CLam*	present (Minas Gerais, Paraná, São Paulo)<br>present (Minas Gerais, Paraná, São Paulo)	*Diaphorina citri*	present (Amazonas, Bahia, Ceará, Pará, Pernambuco, Rio de Janeiro, Santa Catarina, São Paulo)
Columbia	*CLas*	present (few occurrence)	*Diaphorina citri*	widespread
Paraguay	*CLas.*	restricted distribution	*Diaphorina citri*	restricted distribution
Uruguay	-	-	*Diaphorina citri*	few occurrences
Venezuela	-	-	*Diaphorina citri*	restricted distribution
**Europe**				
Portugal	-	-	*Trioza erytreae*	restricted distribution
Spain	-	-	*Trioza erytreae*	restricted distribution
**Oceania**				
American Samoa	-	-	*Diaphorina citri*	present
Guam	-	-	*Diaphorina citri*	present
Papua New Guinea	*CLas*	restricted distribution	*Diaphorina citri*	restricted distribution

Leg.: *C*Las: *Candidatus* Liberibacter asiaticus; *C*Laf: *Candidatus* Liberibacter africanus; *C*Lam: *Candidatus* Liberibacter americanus.
Ref. : CABI (2017), EPPO (2017).
